# Acceptability and utility of, and preference for wearable activity trackers amongst non-metropolitan cancer survivors

**DOI:** 10.1371/journal.pone.0210039

**Published:** 2018-12-31

**Authors:** Sarah J. Hardcastle, Maddison Galliott, Brigid M. Lynch, Nga H. Nguyen, Paul A. Cohen, Ganendra Raj Mohan, Niloufer J. Johansen, Christobel Saunders

**Affiliations:** 1 School of Psychology, Curtin University, Perth, WA, Australia; 2 School of Medicine, University of Western Australia, Crawley, WA, Australia; 3 Cancer Council Victoria, Melbourne, VIC, Australia; 4 St John of God Hospital, Subiaco, WA, Australia; 5 Hollywood Private Hospital, Nedlands, WA, Australia; Universiteit van Amsterdam, NETHERLANDS

## Abstract

**Purpose:**

The study purpose was to investigate the acceptability and utility of, and preference for, wearable activity trackers (WATs) amongst cancer survivors living in regional and remote areas of Western Australia.

**Methods:**

Twenty participants were recruited (Mean age = 63 years, SD = 13) to test two to three trackers from five available models (Fitbit Alta, Garmin Vivofit 2, Garmin Vivosmart, Polar loop 2 and Polar A300). Participants wore each device for two weeks, followed by a one-week washout period between devices. Interviews were conducted with participants to explore user perceptions and experiences. Interview transcripts were analysed using thematic analysis.

**Results:**

Four main themes emerged: (i) Consciousness raising; (ii) Prompts and Feedback; (iii) Accuracy and registry of activities; and, (iv) WAT preferences and features.

**Conclusions:**

WATs were acceptable and useful to cancer survivors. WATs increased self-awareness of physical activity, provided real time feedback in relation to step goals, and reinforced progress and efforts towards goals. The aesthetics of the WATs were deemed crucial in determining preference and likelihood of use.

**Implications for cancer survivors:**

Future interventions may do well to have two different WATs available for participants to choose from, according to activity preferences, aesthetic preferences, and display size.

## Introduction

Cancer is a leading cause of disease burden worldwide [1]. Effective physical activity (PA) interventions to curb the growth in inactivity and prevent chronic illness in cancer survivors are essential [2–3]. PA prevents cardiovascular disease (CVD) [4–5] and can reduce the risk of cancer recurrence [6–9] and improve overall survival, yet few survivors meet current PA guidelines of at least 150-minutes per week of moderate-intensity exercise [10]. Furthermore, there are significant geographic inequalities in cancer survival, which need to be addressed [11]. Effective, distance-based PA interventions are essential in order to reduce such geographic inequalities in survival.

Evidence from trials support the efficacy of PA interventions in producing short-term, but not long-term change in cancer survivors [12,13]. Most interventions tend be supervised and/or facility-based, and subsequently resource-intensive. Home-based and distance-based interventions (such as those using wearable activity technology) may mitigate access and transport issues, and are less expensive than supervised, facility-based programs requiring participants to attend classes or maintain a health club membership [14]. There is a current gap in the literature on less intensive home-based interventions that could be cost-effective, and more scalable.

Interventions that offer opportunities for self-monitoring have successfully increased PA in cancer survivors [13], and survivors report that self-monitoring is a helpful tool for improving PA [15,16]. Wearable activity trackers (WATs) hold great potential as a low-cost self-monitoring tool to increase PA. Lyons et al [17] reviewed 13 different WATs and their associated apps, and concluded that they use many behavior change techniques employed in typical PA interventions (i.e. self-monitoring, feedback, goal-setting). WATs are perceived as useful and acceptable to individuals with chronic conditions [18]. Thus, WATs may represent a relatively low-cost, feasible and scalable approach for widespread PA promotion in cancer survivors.

To date, only a few studies have examined the acceptability and usefulness of WATs to promote physical activity amongst older adults or those with chronic diseases, [18–21] and only one study has explored acceptability in breast cancer survivors [22]. This found participants mostly increased their awareness of the amount of time spent active or number of steps achieved. However, the study recruited only metropolitan breast cancer survivors, and half were already active.

Little is known about the feasibility of using WATs in interventions with survivors, and in particular, to promote PA to regional and remote survivors. In order to design and develop effective interventions, it is essential to first understand the utility of, and preference for WATs, and WAT features amongst such survivors. The aim of this study is to explore the acceptability and utility of, and preference for WATs in cancer survivors living in regional and remote areas of Western Australia, in order to develop more effective interventions.

## Methods

### Participant recruitment

Following hospital HREC approval (St John of God Healthcare # 1157) participants were identified from the databases of breast and gynaecologic oncologists in Perth, Australia and from a list of patients who expressed their interest in future research from previous studies (colorectal and endometrial). Patients were eligible to participate if they: (i) had completed active treatment for cancer within the preceding five years and deemed to be in remission; (ii) were insufficiently physically active (i.e., not meeting the recommended 150-minutes of moderate-intensity PA per week [10]; (iii) resided in a regional and remote areas of WA [23] (i.e., with a postcode of 6200+); and, (iv) had daily access to a handheld device or personal computer and internet. Exclusion criteria were: (i) age <25 and >90 years; (ii) already sufficiently physically active; (iii) currently using a wearable tracker; (iv) inability to comprehend English.

Patients meeting the inclusion criteria (N = 77) were invited to participate by their treating oncologist via mail. Patients registered their interest in participating in the study via email or telephone, and subsequently received a telephone call from a research assistant (RA) to arrange a convenient time to conduct a telephone screening questionnaire to determine PA status and eligibility using the Active Australia questionnaire [24]. Participants provided written consent and gave permission for the interviews to be audio-recorded. They were informed that pseudonyms would be used in reporting of data.

### Procedure

The procedure was based on a previous study by co-authors [22] and involved using five commercially available WATs to assess acceptability and feasibility of wearable technology to increase PA. The selected devices were chosen on the basis that they: (i) included a step-count function; (ii) included a non-movement notification; (iii) had an associated app; and, (iv) were affordable (less than $300AUD). Based on these criteria, five devices were selected for testing: Fitbit Alta, Garmin Vivofit2, Garmin Vivosmart, Polar Loop2 and Polar A300. The selected devices had a range of different features. For example, the Garmin Vivofit 2 used a long life battery rather than needing to be charged; the Garmin Vivosmart measured stairs climbed and the Fitbit had a fixed goal of 10,000 steps.

Participants were assigned two of the five WATs to wear for a minimum of four weeks, two weeks per device. A one-week non-wear ‘wash out’ period took place between trackers. Some participants wore a third device, if time permitted and they were interested in trialling a third device. The expectation was that two wearable trackers would be trialled. A RA mailed written instructions, alongside the tracker concerning how to initialise their tracker and install the app on their smartphone. Participants were also given brief information on how to operate the tracker, its basic features and its app. Tracker features are found in Table 1.

**Table 1:** Specific features of wearable activity trackers selected for testing in the study.

Specific features	Fitbit Alta	Garmin Vivofit 2	Garmin Vivosmart	Polar Loop 2	Polar A300
Non-movement notification	Visual/vibration	visual/ audio	visual/ vibration	Sound/vibration	Sound/vibration
Display steps	✓	✓	✓	✓	✓
Display proportion of steps taken and target goal	✓	✓	✓	✓	✓
Default or auto goal^a^	Default	Auto	Auto	Auto	Default
Altimeter (stairs)			✓		
Heart Rate			✓		
Water resistant (up to 30 metres)		✓	✓	✓	✓
Sleep monitor	✓	✓	✓	✓	✓
Display Size (mm)	25.5 x 10	25.5 x 10.0	25.3 x 10.7	5 x 17	27.5 x 27.5
Cost (AUD)	150	80	189	119	149
Battery life	7 days	1 year	7 days	21 days	4 weeks
**Number of participants testing the device**	12	7	8	6	5

^**a**^ *Default or auto-goal*: “Default” indicates that devices have default step goals of 10,000 steps per day.

“Auto goal” indicates that devices will create a daily step goal automatically based on user’s previous activity levels, i.e., if user achieved only 5000 steps under the goal of 7,500 steps, the step goal will reduce to 6,500 steps on the next day to make the goal more achievable.

#### Data collection

Semi-structured interviews lasting up to 60-minutes were conducted. Interviews took place either at the participant’s home, cafe, or via telephone. S1 Fig provides an overview of the interview guide used with questions concerning experiences, acceptability and preferences of WATs. Questions also explored the utility of devices to increase PA and/or reduce sedentary behaviour (SB) and comfort with technology. Interviews were digitally recorded and transcribed verbatim. Data collection ceased at the point when no new information was gained and data saturation was reached [25]. The study adhered to the consolidated criteria for the reporting of qualitative research (COREQ) [26].

### Data analysis

Data were analyzed using thematic analysis [27]. Thematic analysis involved several steps. The first step involved *immersion* and involved carefully reading transcripts several times to identify participants’ meanings. The second step involved attaching codes to salient text segments. The third step involved the identification of themes at a broader level and examining whether codes may be combined to form an overarching theme. The final step involved reviewing themes, cross-checking for overlap and differences and finally defining and classifying themes. To broaden data interpretation, a second researcher read and coded all transcripts and met with the first researcher to agree on coding and finalise themes. The analysis offered is one interpretation of the interviewees’ experiences and we acknowledge that other interpretations are possible. Nevertheless, we aim to offer a credible and trustworthy interpretation that captures participants’ perceptions and experiences. For example, we provide ‘thick description’ via the use of extensive and direct quotations so that the reader can evaluate the interpretation [28].

## Results

Seventy-seven cancer survivors were invited to participate. Of these seven (9%) expressed interest but were ineligible. Twenty survivors participated in the trial (26%). Sixteen (14 female and 2 male) completed the study with a mean age of 63 (SD 13) years. There were no significant differences in age (*t* (73) = 1.29, *p* = 0.20) and months since diagnosis (*t* (73) = -0.43, *p* = 0.67) of participants that completed the study compared to those who declined participation or dropped out. Participant demographics and study tracker allocation are summarized in Table 2 and Table 3 respectively.

**Table 2:** Participant characteristics.

Age (years)	
Mean	62.58
SD	12.92
Range	35–78
Marital Status	
Married/living together	13
Separated	2
Not married	1
Highest completed education	
Primary	1
Secondary/high school	1
Post-school vocational	6
University Not reported	4 4
Annual household income	
<AUS$ 20,000	1
AUS$ 20,001–$ 39,999	2
AUS$ 40,000–$ 59,999	4
AUS$ 60,000–$ 79,999	1
AUS$ 80,000 +	3
Not reported	5
Employment	
Full-time	6
Part-time	3
Retired	6
Homemaker	1

**Table 3:** Overview of participants and wearable activity trackers trialed.

Participant	Age	Cancer Type	Treatment	1^st^ tracker	2^nd^ tracker	3^rd^ tracker
1	50–59	Uterine	SU+CH+RA	Garmin Vivofit 2	Polar Loop 2	Garmin VivoSmart
2	50–59	Colorectal	SU+CH	Garmin VivoSmart	Polar A300	Fitbit Alta
3	60–69	Colorectal	SU+CH	Garmin VivoSmart	Polar Loop 2	N/A
4	60–69	Breast	SU+CH+RA	Garmin VivoFit 2	Polar Loop 2	Garmin VivoSmart
5	60–69	Colorectal	SU	Garmin VivoSmart	Fitbit Alta	Polar A300
6	50–59	Ovarian	SU+CH	Fitbit Alta	Garmin VivoFit 2	N/A
7	40–49	Breast	SU+CH+RA	Fitbit Alta	Garmin VivoSmart	N/A
8	60–69	Breast	SU+CH	Garmin VivoFit 2	Fitbit Alta	Polar Loop 2
9	70–79	Breast	SU+CH+RA	Garmin VivoSmart	Polar A300	N/A
10	50–59	Breast	SU+CH+RA	Fitbit Alta	Polar Loop 2	N/A
11	70–79	Breast	SU+RA+HR	Polar Loop 2	Fitbit Alta	Garmin VivoFit 2
12	60–69	Colorectal	SU	Fitbit Alta	Polar A300	N/A
13	30–39	Breast	SU+CH	Fitbit Alta	Polar Loop 2	Garmin VivoFit 2
14	50–59	Breast	SU+CH	Fitbit Alta	Garmin VivoFit 2	N/A
15	50–59	Endometrial	SU+CH+RA	Fitbit Alta	Polar A300	N/A
16	60–69	Endometrial	SU+RA	Garmin VivoSmart	Fitbit Alta	N/A

*Note*. *SU = Surgery; CH = Chemotherapy; RA = Radiotherapy; HR = Hormone Therapy*

Analysis of the data identified four main themes: *Increasing Self-Awareness of PA and SB; Prompts and Feedback; Accuracy and registry of activities; WAT preferences and features*. Several participants purchased (n = 5) their own WAT following participation in the study and several others (n = 8) were considering purchasing one. Pseudonyms are used throughout the manuscript. A summary of the themes and illustrative quotes can be found in Table 4.

**Table 4:** Overview of themes and illustrative quotes.

Theme	Brief description	Illustrative quotes
Increasing self-awareness of PA and SB	WATs increased participant self-awareness of their SB and PA.	“It made me get out…take the dog for a walk…walk around the yard (to) get my steps up” (P6, aged 50–59) “Knowledge is power, so it’s the reality of it that you might think you’re doing a certain amount of activity but having it written down helps” (P1, aged 50–59) “The main benefit was it tells you how many steps you’ve done so it’s like quantifying how much exercise that we’re getting on a daily basis” (P2, aged 50–59) “I get home and I’ve only done 3000 steps for the whole day which obviously isn’t enough so it makes you more conscious to keep moving and try move a bit more” (P10, aged 50–59)
Prompts and Feedback	The most motivational dimensions of the WATs was the provision of *prompts* and *feedback*.	“The beep makes you look at the watch and then it tells you it’s time to move…I thought ok I’ll go get a drink of water or something” (P5, aged 60–69) “If I’m watching telly and the ads come on I get up off the chair around the lounge, down the passage through the kitchen to increase my steps, if I didn’t have that on I wouldn’t keep doing it” (P11, aged 70–79) “It prompted me to go for a bit extra walking” (P16, aged 60–69) “Fitbit sends you a weekly report…fireworks on the screen when you reached your target” (P15, aged 50–59)
Accuracy and Registry of Activities	The accuracy of statistics (primarily step count) was questioned by several participants, mainly with reference to the Fitbit which had a tendency to over count steps during sedentary or light activity. Others expressed disappointment because the WATs did not record other activities such as stair climbing, swimming, gardening or cycling.	“The Fitbit needs to be made more accurate, it’s giving people a false sense of achievement” (P14, aged 50–59) “I did an hour of yoga but it didn’t register that” (P8, aged 60–69) “The first one was really nice except, that I climbed up all these stairs deliberately because I wanted it recorded. And I did four flights of, five flights of stairs and it recorded one (Garmin Vivosmart) (P3, aged 60–69) “Digging up a hole, large tree stump shurbs…didn’t record as an activity…you might get a little disillusioned, what have I got to do to get an activity recorded” (P4, aged 60–69)
WAT Preferences: Appearance & Functionality	Appearance and functionality were commonly cited in relation to acceptability and usability. Most participants preferred WATS with a slim band.	“The Fitbit is quite difficult to put on” (Miriam, aged 67); “I much preferred the Fitbit because it was smaller and easier to work with” (P12, aged 60–69). “This one (Fitbit) was smaller, less bulky and easier to see” (P10, aged 50–59) “This is a cute little one (Garmin Vivofit), it fits my wrist perfectly…you don’t even have to charge it” (P11, aged 70–79)
WAT Preferences and Features: Preferred Features	The most popular feature was step counting. Several wanted the WAT to have a heart rate monitor. WATs that were easy to sync were also viewed favourably.	“All I ever focused on was the step count” (P5, aged 60–69); “I used them for steps mainly” (P15, aged 50–59) “But I like the heart rate monitor on that one [Garmin] which I thought was good” (P2, aged 50–59); “The syncing was a pain on that one (Garmin Vivosmart) but the tapping (Fitbit) was also a pain” (P6, aged 50–59) “With the Garmin, I didn’t get on that well with it, couldn’t work out a lot of things to do with it, with the Fitbit I was able to download the app and follow it a bit more…it records the calories and steps” (P16, aged 60–67)

### Increasing self-awareness of PA and SB

Most participants reported that the trackers made them more conscious of their PA and SB: “they motivated me…made me very aware of how much I’m moving and that I need to move more” (Lisa, aged 49). Self-awareness of PA was viewed as important and the WATs provided this awareness: “I think it helps to know how much you’re doing, what you’re doing” (Emma, aged 50) and “It certainly prompts my mind to think you’re not doing enough” (Sandra, aged 68). Several participants expressed surprised at the effort needed to reach the recommended steps: “If you’re doing something very stationery I was surprised that you didn’t get much of a count” (Paul, aged 52) and “makes you more aware because I have an office job …you don’t take as many steps during those days” (Anna, aged 35). Awareness of SB was also identified: “just look at how much time you spent sitting” (Diane, aged 67) and “I’ve got to move because this thing’s telling me I’ve been sitting too long” (Elsa, aged 78). In recognition of negative cases, only one participant did not enjoy using the WATs: “I was always worried about steps, steps, steps…you’ve got to do steps. It’s intrusive on my life” (Miriam, aged 67).

### Prompts and feedback

The provision of prompts and feedback were the most motivational aspects of the WATs. The WATs acted as a prompt: “yeah it prompted me to go…like I could have gone up the escalator but I elected to go up the stairs…and then I was cross because it didn’t show (record stairs climbed)” (Carol, aged 68) and for Paul “it prompted me to do it…I knew that if I went for a decent walk then I’d always get the 10000” (Paul, aged 52). Real time feedback was important to nudge behaviour and meet goals: “Geez I haven’t done enough sometimes at night I’m 500 short so I go outside in the dark and check on the animals to get my 10000” (Karen, aged 51).

Participants also found the automated prompts to move motivating: “every time it buzzed, you got up and moved so you feel great success” (Diane, aged 67) and “last night I looked—ahh that’s the red mark!, so I jumped off the chair and round I went…if I didn’t have that on I wouldn’t keep doing it” (Elsa, aged 79). However, several participants found these reminders too simplistic and in the case of the Garmin’s ‘MOVE!’ prompt, rude: “It buzzed at you and went MOVE … even had an exclamation mark, it’s like ‘seriously’ you get slightly offended” (Emma, aged 50). For Paul, these prompts were rather ‘Pavlovian’; he offered his thoughts on what may work:

“Quantitative data are good because you’re reminded that you’re not meeting your targets…if set at 10,000 and you get 7,000 for a couple of days then maybe you could get direct messages, bit of a psych talk…automated but make it personalised, you missed your target for a few days rather than saying Move!”

Several participants also referred to the feedback and praise concerning achievements as important and motivational: “very motivational feedback…computer graphics, gave you your percentage ranking for the day so you could see how you were getting better” (Diane, aged 67, referring to Polar A300) and “all of them had a goal for the day…the step goal was good…it said you’d done over 200% of your step goal” (Maureen, aged 65).

### Accuracy and registry of activities

The accuracy of statistics was identified as a theme and related predominantly to the accuracy of the Fitbit which had a tendency to count arm movements as steps: “Watch this (shakes arm) I said what’s the number and I went like this (shakes arm) and she went oh…it can be tricked” (Maureen, aged 65) and “I’m not sure I believe them, it’s quite easy to get the steps in….washing dishes or sanding something down, I tended to get the 10,000 steps pretty easy” (John, aged 69). The Polar A300 also misreported activity: “My hand was moving for a couple of minutes, it thought I was running” (Diane, aged 67).

The inaccuracy of the Fitbit led to a false sense of achievement for some. For example, Linda found that it was relatively easy to accumulate steps: “I cleaned the house on Saturday and I did 9,800 steps just cleaning…it doesn’t take much at all (Linda, aged 51). For others, there was disappointment because the WATs didn’t record other activities such as swimming: “it doesn’t take into account other exercise like swimming” (Miriam, aged 67), cycling: “I found (cycling) a bit tricky to track so I put in the kilometers in an area of the App” (Lisa, aged 49) or gardening: “It doesn’t record gardening at all” (Maureen, aged 65).

### WAT preferences and features

WAT preferences and features contain two sub-themes: appearance and functionality, and preferred features.

#### Appearance and functionality

Appearance was a commonly cited factor in relation to acceptability. The most aesthetically pleasing WAT was the Fitbit because of its slim band and small face: “I thought that one was quite sleek… not overbearing or overstated” (Paul, aged 52) and “from an aesthetic point of view it’s more stylish” (Fiona, aged 50). Participants also liked the appearance of the Garmin VivoSmart (“I like this watch…the face stays on” (Lisa, aged 49) and VivoFit, as they were both slim in appearance. The Polar Loop was also popular in appearance but perceived as uncomfortable since it’s more like a bangle and would slide around. The Polar A300 was not visually appealing and often described as bulky and clunky: “The polar was too big on, too clunky so I took it off” (John, aged 69).

The most common complaint regarding useability concerned the Fitbit. Participants often spoke about experiencing difficulties putting the band on: “I physically could not press those metal things through so I had to have my husband put it on” (Carol, aged 68) and problems looking at the statistics: “I kept tapping and tapping and nothing would happen” (Miriam, aged 67) and “you had to find x marks the spot” (Elsa, aged 78). The Garmin VivoFit was described with differing opinions. Some participants liked the security of the clip to lock in the band, whilst others found it awkward and annoying: “that was far too hard to do it yourself…that was one that was two little hooks in” (Maureen, aged 65). The Garmin Vivosmart was popular because it had a normal watch strap: “I’m seriously thinking of getting a Garmin (vivosmart)…I like the fact it had a proper strap” (Carol, aged 68), and displayed clear text: “For an older person, I would give them the Garmin because of its bigger writing and more accurate” (Karen, aged 51).

There were few issues reported concerning limited tech-knowledge even amongst the older participants: “pretty straight forward…for a techo nerd” (Carol, aged 68) and “I’ve got the piece of paper sat down, got my phone out, had to press the button, hang onto it until the word came up, perfect” (Elsa, aged 78). Only one participant did not understand how to use them: “I’m not very computer literate…I couldn’t get it to work” (Sue, aged 78). All participants engaged with the Apps apart from Elsa and Sue. Elsa was content using solely the device to self-monitor her activity and Sue was unable to set up either device.

#### Preferred features

The most popular feature across all WATs was the step counting which promoted self-awareness. Most participants’ desired simple features as summarised by Elsa “What time it is, how many steps I’ve done, how far I’ve gone, that’s all I wanted” (aged 78). Most participants found the automated prompts to increase step count as motivational, however the tone of the prompt was important. The tone of the Garmin was perceived as abrupt and rude whereas the tone of the Fitbit was experienced as more polite: “I’d get a little vibration to say let’s go do 250 steps, it was much more polite than MOVE” (Fiona, aged 50).

Trackers that were easy to sync (i.e., Fitbit) were also viewed favorably. There was also a preference for the Fitbit app: “Fitbit was superior for quick syncing and notifications associated with the App…had a lot more versatility…it was on autosync…the Garmin you had to force sync” (Fiona, aged 50), and, “Garmin was a bit more complex to set up…even with the food/ drinking you had to get a different app called Myfitnesspal” (Lisa, aged 49).

The less preferred features have been highlighted and include issues relating to the accuracy of trackers and disappointment that WATs did not effectively capture all activities. For some, the auto-goal function on some trackers caused confusion: “it wasn’t clear like now the goal, does that mean I have to do 5000 steps, why have they got that I’ve been doing over 10000” (Elsa, aged 78). Despite the study recruiting regional and remote survivors, there were very few concerns regarding internet availability. Only Anna mentioned this: “The internet and phone coverage is bad…that is a barrier if you want to download apps…sometimes you have to use mobile data to look at your statistics”.

## Discussion

This is the first study to explore the experiences, acceptability and usability of WATs amongst rural cancer survivors. Despite the evidence of the importance of PA for healthy survivorship [4–9], few survivors meet the PA guidelines [10]. As a powerful self-monitoring tool, WATs present a potential opportunity for PA promotion and in particular, to reach survivors living in rural communities. WATs may also play a role in objectively monitoring patient PA remotely and used to predict important clinical outcomes such as readmission. A recent study found that a higher mean daily step count, derived from the Fitbit, predicted lower risk of readmission following metastatic peritoneal cancer [29]. In addition to the use of WATs to promote PA to survivors following active treatment to prevent CVD, WATs could also play an important role in identifying opportunities for real-time adaptive interventions during cancer treatment to improve clinical outcome [29, 30].

We found that WATs increased self-awareness of their PA and SB consistent with previous research [18,20]. The provision of prompts and feedback were the most motivational aspects of the WATs. The trackers provided continuous self-monitoring and real time feedback in relation to step goals and appeared to reinforce progress or nudge behaviour towards goals. Recognition of achievements (either from the device or through the App) were also enjoyed by participants. The motivational nature from immediate feedback on step count and progress towards step goals has been reported previously [20]. The features of WATs that most motivated participants (i.e., monitoring behavior, prompts, setting goals, receiving feedback) are also behavior change techniques known to be effective for promoting PA [31,32]. For example, ‘provide instruction’ and ‘reinforcing efforts towards behavior’ are techniques associated with a significant increase in PA [32]. Interventions that employ self-monitoring and at least one other self-regulatory technique (i.e., goal setting, receiving feedback on performance, review of goals) are significantly more effective in promoting PA [31].

The majority of participants found the WATs to be highly acceptable and useful consistent with previous research in older populations, [19,21] chronic disease [18,20] and breast cancer survivors [22]. This is the first study to indicate that the provision of written instructions and/or telephone technical support in conjunction with WATs may be sufficient in rural survivors and that such interventions do not require face-to-face contact. Only one participant reported finding the devices intrusive and one other who was unable to use the technology.

Some were sceptical of the authenticity of the Fitbit-recorded data, noting that it tended to over-count steps. Others failed to notice the devices misclassifying steps during SB or light activity leading to a false sense of achievement. However, research supports the accuracy of WATs, including the Fitbit for counting steps [33,34]. The reliability and validity of two Fitbit models (Flex and ChargeHR) has shown to be good in older adults (aged over 65), with high intra class correlations of the WATs with direct observation of steps [35]. Good strength of agreement was also found for total distance and moderate-to-vigorous physical activity in the free-living environment compared to an accelerometer [35]. A further study in older adults also found excellent agreement between the Fitbit (One and Zip) and direct observation for step count, and also between the Fitbit and Actigraph for average step count/per day over 7days [36]. It would appear that Fitbit trackers are sufficiently accurate to be used in community-dwelling older adults to increase physical activity. The over-count of steps found in the present study appears to occur only during light household activities. Therefore, future trials should ensure the device is worn on the non-dominant wrist or taken off when performing stationery activities involving much arm movement. Some participants were disappointed that activities such as swimming and cycling were not recorded by the device. Such frustration that WATs fail to register activities has been noted elsewhere [20]. This is a limitation with some models which are not waterproof and/or do not have more sophisticated features.

In relation to preferences, the aesthetics of the WATs were deemed crucial in determining preference and likelihood of use, consistent with previous research [18, 21,22]. The smaller trackers were mostly preferred with the Fitbit Alta being considered the most stylish. The Garmin models were also favourable due to their slim in appearance and because the screen was bigger and the display stayed on. The most common complaint regarding useability concerned the Fitbit Alta and difficulties tapping to view the statistics. The acceptability of WATs is highly influenced by device characteristics such as display, comfort and most of all aesthetics.

The most preferred feature across WATs was step counting and this is consistent with the previous study in breast cancer survivors [22]. The prompts were deemed helpful although the tone of the prompt was considered important. The Fitbit appeared to be the preferred App; participants found it more intuitive and some were interested in self-monitoring food intake, which were not available on other Apps.

### Clinical implications

The WATs provided self-monitoring and real time feedback in relation to step goals and reinforced progress and efforts towards goals. Future interventions may do well to have two different WATs available for participants to choose from, according to activity preferences, aesthetic preferences, and display size.

### Strengths and limitations

Our study has certain limitations including a low response rate, which may have introduced response bias. Additionally, our sample is predominantly female (87.5%) (since patient enrolment was limited to databases with a heavy preponderance of female malignancies including breast and gynecologic). Therefore we cannot assume that our findings are transferable to male cancer survivors, particularly the findings in relation to aesthetic preferences. Further, extended-term acceptance could not be explored. A study strength is the recruitment of underserved survivors living in rural areas, and the first feasibility testing of providing WATs with written instructions and/or telephone technical support.

## Conclusion

WATs appear to be acceptable to cancer survivors living in rural locations and may represent a low-cost, feasible and scalable approach for PA promotion. WATs increased self-awareness of physical activity, provided real time feedback in relation to goals, and reinforced progress and efforts toward goals. The aesthetics of the WATs were deemed crucial in determining likelihood of use. Future interventions may do well to have two different WATs available for participants to select from, according to activity preferences, aesthetic preferences and display size. Future research should explore whether WAT use and increased PA is sustained over time.

## Supporting information

S1 Fig Interview guide. The interview guide provides examples of questions used during interviews concerning experiences, acceptability and preferences of WATs.
(PDF)

## References

[1] International Agency for Research on Cancer:World Cancer Report 2014. http://publications.iarc.fr/Non-Series-Publications/World-Cancer-Reports/World-Cancer-Report-2014.

[2] Friedenreich CM, Neilson HK, Farris MS, & Courneya KS. Physical activity and cancer outcomes: A precision medicine approach. *Clin Cancer Res*. 2016;22: 4766–4775. 10.1158/1078-0432.CCR-16-0067 27407093

[3] Keats MR, Cui Y, Grandy SA, Parker L. Cardiovascular disease and physical activity in adult cancer survivors: A nested, retrospective study from the Atlantic PATH cohort. *J Cancer Surviv*. 2017; 264–273. 10.1007/s11764-016-0584-x 27854007

[4] Stewart J, Manmathan G, Wilkinson P. Primary prevention of cardiovascular disease: A review of contemporary guidance and literature. JRSM Cardiovasc Dis. 2017; 6:2048004016687211 10.1177/2048004016687211 28286646 PMC5331469

[5] Sattelmair J, Pertman J, Ding EL, Kohl HW, Haskell W, & Lee IM. Dose response between physical activity and risk of coronary heart disease: A meta-analysis. *Circulation*. 2011;124: 789–795. 10.1161/CIRCULATIONAHA.110.010710 21810663 PMC3158733

[6] Meyerhardt JA, Heseltine D, Niedzwiecki D, Hollis D, Saltz LB, Mayer RJ et al Impact of physical activity on cancer recurrence and survival in patients with stage III colon cancer: Findings from CALGB 89803. *J Clin Oncol*. 2006;24: 3535–3541. 10.1200/JCO.2006.06.0863 16822843

[7] Friedenreich CM, Wang Q, Neilson HK, Kopciuk KA, McGregor SE, Courneya KS. Physical activity and survival after prostate cancer. *Eur Urol*. 2016;70: 576–585. 10.1016/j.eururo.2015.12.032 26774959

[8] Dieli-Conwright CM, Lee K, Kiwata JL. Reducing the risk of breast cancer recurrence: An evaluation of the effects and mechanisms of diet and exercise. *Curr Breast Cancer Rep*. 2016;8: 139–150. 10.1007/s12609-016-0218-3 27909546 PMC5112289

[9] Hamer J, Warner E. Lifestyle modifications for patients with breast cancer to improve prognosis and optimize overall health. *CMAJ*. 2017;189: E268–E274. 10.1503/cmaj.160464 28246240 PMC5318212

[10] Rock CL, Doyle C, Demark-Wahnefried W, Meyerhardt J, Courneya KS, Schwartz AL, et al Nutrition and physical activity guidelines for cancer survivors. *CA Cancer J Clin*. 2012;62: 243–274. 10.3322/caac.21142 22539238

[11] Tervonen HE, Aranda S, Roder D, You H, Walton R, Morrell S, et al Cancer survival disparities worsening by socio-economic disadvantage over the last three decades in New South Wales, Australia. B*MC Public Health*. 2017;17: 691–696. 10.1186/s12889-017-4692-y 28903750 PMC5598077

[12] Goode AD, Lawler SP, Brakenridge CC, Reeves MM, & Eakin EG. Phone, print and web-based interventions for physical activity, diet and weight control among cancer survivors: a systematic review. *Journal Cancer Surviv*. 2015;9: 660–82. 10.1007/s11764-015-0442-2 25757733

[13] Bluethmann SM, Vernon SW, Gabriel KP, Murphy CC, Bartholomew LK. Taking the next step: a systematic review and meta-analysis of physical activity and behavior change interventions in recent post-treatment breast cancer survivors. *Breast cancer research and treatment*. 2015;149: 331–342. 10.1007/s10549-014-3255-5 25555831 PMC4310805

[14] Hardcastle SJ, & Cohen PA. Effective Physical Activity Promotion to Survivors of Cancer Is Likely to Be Home Based and to Require Oncologist Participation. *J Clin Oncol*. 2017;35: 3635–3637. 10.1200/JCO.2017.74.6032 28915086

[15] Hardcastle SJ, Glassey R, Salfinger S, Tan J, Cohen PA. Factors Influencing Participation in Health Behaviours in Endometrial Cancer Survivors. *Psycho-Oncology*. 2017;26: 1099–1104. 10.1002/pon.4288 27665487

[16] Hardcastle SJ, Maxwell-Smith C, Zeps N, Platell C, O'Connor M, & Hagger MS. A Qualitative Study Exploring Health Perceptions and Factors Influencing Participation in Health Behaviors in Colorectal Cancer Survivors. *Psycho-Oncology*. 2017;26: 199–2015. 10.1002/pon.4111 26935994

[17] Lyons EJ, Lewis ZH, Mayrsohn BG, & Rowland JL. Behavior change techniques implemented in electronic lifestyle activity monitors: A systematic content analysis. *Journal of Medical Internet Research*. 2014;16: e192 10.2196/jmir.3469 25131661 PMC4147713

[18] Mercer K, Giangregorio L, Schneider E, Chilana P, Li M, Grindrod K. Acceptance of commercially available wearable activity trackers among adults aged over 50 and with chronic illness: a mixed-methods evaluation. *JMIR mHealth and uHealth*. 2016; 4:1. 10.2196/mhealth.4225 PMC4749845 26818775

[19] McMahon SK, Lewis B, Oakes M, Guan W, Wyman JF, Rothman AJ. Older Adults’ Experiences Using a Commercially Available Monitor to Self-Track Their Physical Activity. *JMIR mHealth and uHealth*. 2016;4:2. 10.2196/mhealth.5120 PMC4848389 27076486

[20] Gualtieri L, Rosenbluth S, Phillips J. Can a free wearable activity tracker change behaviour? The impact of trackers on adults in a physican-led wellness group. *JMIR Res Protoc*. 2016;5(4):e237 10.2196/resprot.6534 27903490 PMC5156822

[21] Puri A, Kim B, Nguyen O, Stolee P, Tung J, Lee J. User acceptance of wrist-worn activity tracker among community-dwelling older adults: Mixed method study. *JMIR Mhealth Uhealth*. 2017;5:e173 10.2196/mhealth.8211 29141837 PMC5707431

[22] Nguyen NH, Hadgraft NT, Moore MM, Rosenberg DE, Lynch C, Reeves MM, et al A qualitative evaluation of breast cancer survivors’ acceptance of and preferences for consumer wearable technology activity trackers. *Supportive Care in Cancer*. 2017;25: 3375–3384. 10.1007/s00520-017-3756-y 28540402

[23] ASGS 2011 Australian Statistical Geography Standard, Remote areas. Canberra, AIHW.

[24] Brown WJ, Burton NW, Marshall AL, & Miller YD. Reliability and validity of a modified self-administered version of the Active Australia physical activity survey in a sample of mid-age women. *Australia and New Zealand Journal of Public Health*. 2008;32: 535–41. 10.1111/j.1753-6405.2008.00305. 19076744

[25] Sparkes AC, Smith B. Qualitative research methods in sport, exercise and health. Routledge: London, 2014.

[26] Tong A, Sainsbury P, Craig J. Consolidated criteria for reporting qualitative research (COREQ): a 32-item checklist for interviews and focus groups. *International Journal for Quality in Health Care*. 2007;19: 349–357. 10.1093/intqhc/mzm042 17872937

[27] Braun V, Clarke V. Using thematic analysis in psychology. *Qual Res Psychol*. 2006;3: 77–101. 10.1191/1478088706qp063oa

[28] Hardcastle SJ, & Hagger MS. "You can't do it on your own": Experiences of a motivational interviewing intervention on physical activity and dietary behaviour. *Psychology of Sport and Exercise*. 2011;12: 314–323.

[29] Low CA, Bovbjerg DH, Ahrendt S, Choudry H, Holtzman M, Jones HL, Pingpank JF, Ramalingam L, Zeh HJ, Zureikat AH, Bartlett DL. Fitbit stepcounts during inpatient recovery for cancer surgery as a predictor of readmission. *Ann Behav Med*. 2018;52: 88–92. 10.1093/abm/kax022 29538623 PMC6051699

[30] Low CA, Dey AK, Ferreira D, Kamarck T, Sun T, Bae S, Doryab A. Estimation of symptom severity during chemotherapy from passively sensed data: Exploratory study. J *Med Internet Res*. 2017;19:e420 10.2196/jmir.9046 29258977 PMC5750420

[31] Michie S, Abraham C, Whittington C, McAteer J, Gupta S. Effective techniques in healthy eating and physical activity interventions: a meta-regression. *Health Psychology*. 2009;28: 690 10.1037/a0016136 19916637

[32] Williams SL, French DP. What are the most effective intervention techniques for changing physical activity self-effifacy and physical activity behaviour- and are they the same? *Health Education Research*. 2011;26: 308–322. 10.1093/her/cyr005 21321008

[33] Stockpool C, Porcari J, Gillette C, Foster C. The accuracy of various activity trackers in estimating steps taken and energy expenditure. *J Fitness Res*. 2014;3: 32–48.

[34] Nelson MD, Kaminsky LA, Dickin DC, Montoye AH. Validity of consumer-based physical activity monitors for specific activity types. *Med Sci Sports Exer*. 2016;48: 1619–1628. 10.1249/MSS.0000000000000933 27015387

[35] Burton E, Hill KD, Lautenschlager NT, Thogerson-Ntoumani C, Lewin G, Boyle E, Howie E. Reliability and validity of two fitness tracker devices in the laboratory and home environment for older community-dwelling adults. *BMC Geriatrics*. 2018;18:103 10.1186/s12877-018-0793-4 29724191 PMC5934836

[36] Paul SS, Tiedmann A, Hassett LM, Ramsay E, Kirkham C, Chagpar S, Sherrington C. Validity of the Fitbit activity tracker for measuring steps in community-dwelling older adults. *BMJ Open Sport Exerc Med*. 2015;0:e000013 10.1136/bmjsem-2015-000013 PMC5117050 27900119

