# Supplementary material for: Acceptability and utility of, and preference for wearable activity trackers amongst non-metropolitan cancer survivors
Source: PLoS One. 2018 Dec 31;13(12):e0210039. doi: 10.1371/journal.pone.0210039 (PMC6312256; doi:10.1371/journal.pone.0210039)
Supplement: S1 Fig — The interview guide provides examples of questions used during interviews concerning experiences, acceptability and preferences of WATs. (PDF) [file pone.0210039.s001.pdf]

SI Fig. Interview Guide

- Please could you describe your experiences using the wearable devices?
- In what ways did you use the wearable devices?
- Were the wearable devices helpful to increase your physical activity level or reduce sedentary behaviour (i.e., time spent sitting down)? If so, in what ways?
- Which aspects did you view or make use of the most? (Note: if participants refer to step count, prompt knowledge and awareness of Moderate-to-vigorous physical activity guidelines and the accumulation of active minutes on the device(s).
- What were the some of the useful aspects of wearing the trackers?
- What were the some of the less useful aspects of wearing the trackers?
- When you wore your physical activity monitor, how did you feel about wearing it?
- What were the features of the way the monitors looked that you most liked and disliked?
- How easy or difficult did you find using the activity devices?
- How easy or difficult did you find downloading or linking the activity monitor to your phone/pc? Did you engage mostly with the device or with the App or both?
- Overall what is your opinion towards activity monitors?
- Which activity monitor would you choose and why?
